# A quantitative analysis of the contribution of melanopsin to brightness perception

**DOI:** 10.1038/s41598-019-44035-3

**Published:** 2019-05-20

**Authors:** Masahiko Yamakawa, Sei-ichi Tsujimura, Katsunori Okajima

**Affiliations:** 1 Graduate School of Environment and Information Sciences, Yokohama National University, Yokohama, Japan; 2 Department of Information Science and Biomedical Engineering, Kagoshima University, Kagoshima, Japan; 3 Faculty of Environment and Information Sciences, Yokohama National University, Yokohama, Japan

**Keywords:** Perception, Human behaviour

## Abstract

In the retina, intrinsically photosensitive retinal ganglion cells (ipRGCs) which express photopigment melanopsin have been identified as photoreceptors which differ from cones and rods. It has been established that such melanopsin-expressing RGCs are involved in the circadian photo-entrainment and pupillary light reflexes. An additional projection from ipRGCs to the lateral geniculate nucleus has been identified, which indicates the association of ipRGCs with visual perception induced by the image-forming pathway. Reportedly, ipRGCs modulate brightness perception but quantitative analysis of brightness perception involving melanopsin and cones-based signals has not been elucidated. We conducted brightness perception experiments that involved melanopsin using a novel projector with six primary colors and formulated the results for melanopsin and cone stimuli. The white visual stimuli (5 degrees in size) that we used had a single xy-chromaticity values but melanopsin stimuli were modulated by designing different spectral distributions. Perceived brightness was measured using a magnitude estimation method at several luminance levels in the near periphery (7 degrees). Additionally, pupil diameter was measured for estimating the intensity of visual stimuli on the retina. The results showed that the perceived brightness of a white visual stimulus with different spectral distributions can be described by a summation of the nearly linear melanopsin response and the non-linear cone response with weighted coefficients, and the contribution ratio of melanopsin in brightness perception increased to 50% and more with increasing visual stimulus. These suggest that melanopsin signals play a crucial role in the estimation of the absolute intensity of the light environment by obtaining absolute brightness information even when cones are adapted by light.

## Introduction

The photoreceptors in the retina that are responsible for forming images typically comprise cones and rods. The light radiation signals received on the retina are encoded and projected to the primary visual cortex (V1) through the retinal ganglion cells (RGCs) and neurons in the lateral geniculate nucleus (LGN) via the chromatic or luminance channels. The RGCs and the LGN neurons respond to the inputs produced in time or in space projected upon their receptive fields^1^. This pathway is called the image forming pathway. Novel photoreceptors known as intrinsically photosensitive RGCs (ipRGCs)^2,3^ have been detected in the retina which express melanopsin^4,5^ and their maximum spectral sensitivity is approximately 480 nm^3,6,7^. In addition, ipRGCs receive inputs from cones and rods^8–10^. IpRGCs are also known to signal to other retinal neurons such as amacrine cells^11–13^. On the other hand, studies have found that light signals received by the melanopsin-expressing RGCs are projected to the suprachiasmatic nucleus (SCN), which entrains circadian rhythms^14^, and to the olivary pretectal nucleus (OPN), which controls the pupillary light reflex^15^. This pathway is called the non-image forming pathway. Further evidence such as the projection to the superior colliculus (SC)^16–18^ and the contribution to migraine-associated photophobia^19,20^ suggests that melanopsin signals are involved in other non-image-forming visual effects as well.

Some studies have indicated the association of melanopsin with the image-forming pathway^8,21–27^. For example, S-off and (L + M)-on signal inputs to ipRGCs and contribute to conscious visual perception by projection to the dorsal LGN^8^. In addition, studies have reported a contribution of melanopsin to neural coding in the thalamus of knockout mice lacking both cones and rods^21^, brightness discrimination ability by melanopsin using the same line of knockout mice both lacking cones and rods^22,23^, an involvement in discrimination by brightness perception by melanopsin signals in human psychophysical research^23^, melanopsin-dependent measure of environment brightness^24^, and responsibility for luxotonic activity^25^. Spitschan *et al*. clarified the relationship between visual stimulation of melanopsin and visual cortex response using fMRI^27^. However, neither the magnitude of melanopsin contribution nor the visual information processing mechanisms, including melanopsin for brightness perception have been clarified.

In this study, we aimed to quantitatively evaluate brightness perception in consideration of melanopsin signals. Isolation of melanopsin functioning from the visual processing by cones and rods is crucial for quantifying the pure contribution of melanopsin in the human visual system. The metamerism method is effective in separating the signals of cones and rods from those of melanopsin where those spectral sensitivities overlap on the wavelength domain. Pupillary light reflex studies^28–30^, a temporal contrast sensitivity study^31^, and a discrimination by brightness perception study^23^ were conducted using this method. White visual stimuli were prepared by designing the spectral power distributions so that only melanopsin stimuli are modulated while the colorimetric values are kept intact. In addition, visual stimuli were presented to a semi-periphery to ensure the contribution of melanopsin. We measured the brightness of each stimulus with a magnitude estimation method. Furthermore, pupil diameter under each visual stimulus was measured for clarifying the relationship between brightness perception and the actual intensity of visual stimulus on the retina.

On the basis on the experimental results, we formulated brightness perception using the stimuli on melanopsin and cones, and the pupil diameter as explanatory variables. These results showed that melanopsin is not a minor contributor in brightness perception but, rather present as a critical factor for the formulation.

## Methods

### Visual stimuli

**Figure 1:**
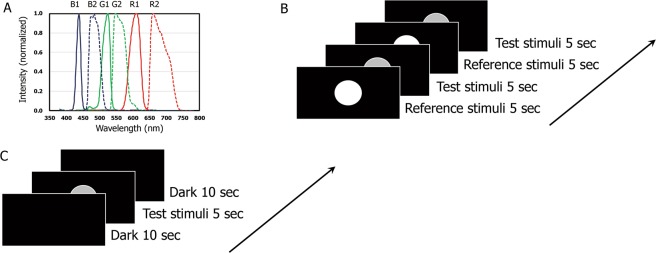
Experimental setup. (**A**) Normalized spectral power distribution of the emission from the 6-color projector. The spectral power distributions of #1 projector are B1, G1, R1 and those of #2 projector are B2, G2, R2. (**B**) Presentation sequence of reference and test stimuli in the measurement of perceived brightness. (**C**) Presentation sequence of test stimuli in the measurement of pupil diameter.

Visual stimuli were prepared by mixing lights emitted from two six-primary-color projectors (DLA–M2000SC; JVC, Yokohama, Japan) with different RGB spectral power distributions^32^ controlled by two personal computers. The spectral power distributions of each primary color are shown in Fig. 1A. The peak wavelengths were 610, 524, 438, 659, 547, and 483 nm and the full widths at half maximum were 37, 26, 15, 61, 42, and 37 nm, respectively. Visual stimuli were prepared on the screen by overlapping the light emission from the two projectors.

**Figure 2:**
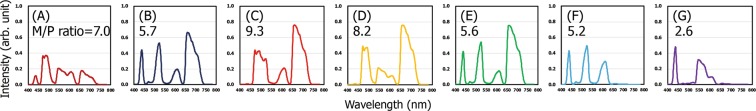
Spectral power distributions of visual stimuli that are metamers of each other. Each stimulus intensity for L-, M-, and S-cones is identical, and each stimulus intensity for rods of (**A**,**B**) is also identical. Numerical values in the figure indicate melanopic/photopic ratio (M/P ratio) defined by $4557\int i(\lambda )P(\lambda )d\lambda /683\int V(\lambda )P(\lambda )d\lambda$. These values represent the stimulus intensity on melanopsin per 1 cd/m^2^.

Four primary colors out of six primary colors were chosen and used as visual stimuli with a constant stimulus intensity on cones. Five primary colors out of six primary colors were used for each stimulus with a constant stimulus intensity on cones and rods. The spectral power distributions were created as per methods by Vienot *et al*.^29^ and Cao *et al*.^30,31^. The cone fundamentals (Stockman & Sharpe, 2000) were used as the spectral sensitivity spectra for L-, M-, and S-cones. The spectral luminous efficiency function for scotopic vision *V’*(*λ*) was used as the spectral sensitivity for rods. For melanopsin stimulation, the lens corrected melanopic spectral sensitivity function proposed by Lucas *et al*.^6,24^ was used. Two types of visual stimuli ((A) and (B) of Fig. 2) with the same stimulus intensity on each of L-, M-, and S-cones and rods were prepared, although the stimulus intensity on melanopsin was different, even at the same luminance. Five types of visual stimuli ((C), (D), (E), (F) and (G) of Fig. 2) with the same stimulus intensity on each of L-, M-, and S-cones were prepared, whereas the stimulus intensity on melanopsin was different even at the same luminance. The reason behind the conditions using visual stimuli with the same stimulus intensity on each of cones and rods was to obtain positive proof that brightness perception in this experiment must be attributable to the involvement of melanopsin, although ipRGCs have inputs from cones and rods. The spectral power distributions of these visual stimuli and the M/P ratio that represents the stimulus intensity on melanopsin at the same luminance conditions is presented in Fig. 2. The M/P ratio was defined as the ratio of the stimulus intensity on melanopsin cells^6^ to the luminance. We calculated the M/P ratio of “Daylight” from low color temperature (2000 K) to high color temperature (7000 K) in the real world to compare with those in this experiment (Fig. S1). Contrast of M/P ratio was maximum 3.6. The luminance of visual stimuli was modulated from 22 to 112 cd/m^2^. The xy-chromaticities of visual stimulation were set to (*x, y*) = (0.328, 0.367), which appeared as a white color. Changes in luminance values were made by controlling the RGB values of the two projectors independently. The chromaticity values, luminance value, and spectral power distribution of visual stimuli were measured by a spectral meter (SR-3A; Topcon Corporation, Tokyo, Japan). The stimulus intensity on cones was adjusted by the photometric value. It was confirmed that there was no difference in L-, M-, and S- cone stimulus intensity among each visual stimulus at the same luminance by calculation using the spectral power distribution of the visual stimulus and the cone spectral sensitivity. The position of the lens of the luminance meter was set to the eye position of the participants for the measurement.

The light from each projector was projected on a white screen set in front of the participants. The test field of view was circular, and the viewing angle was set at 5 degrees. The fixation point was set at 7 degrees from the center of visual stimulation to present stimuli to the nasal side of the peripheral vision of right eye based on the report of maximal melatonin suppression on exposure of the nasal part of the retina by Visser *et al*.^33^. The luminance value on the outside of the circular test field was under the minimum limit of measurement. Participants made to sit in front of and 1.35 m from the screen, and their heads were fixed with a chin rest. Participants observed the visual stimuli reflected on the white screen from the output of the projectors.

### Brightness estimation

Nine (25.0 ± 4.0 years, 7 males and 2 females) and seven (25.1 ± 4.5 years, 5 males and 2 females) Yokohama National University students participated in the measurement of constant cones and rods stimulus intensity experiment, and constant cones stimulus intensity experiment, respectively. Color vision was confirmed to be normal in all participants. This was tested using an Ishihara color plate, a Farnsworth-Munsell 100 Hue test and an anomaloscope. Two participants required vision correction, but we did not use corrective lenses that cause changes in the spectral power distribution of the stimulating light received. According to the Yokohama National University Committee on Life Science Research guidelines, this study protocol was exempted from formal ethics review. All participants consented for the experiments in accordance with Yokohama National University Rules on Life Science Research and provided written certificates of consent. We set rest time in the dark place for 5 minutes for condition leveling, then conducted experiments. For visual stimuli, pairs of the reference stimulus and a test stimulus were used. After the reference stimulus with maximum stimulus intensity on melanopsin was presented for 5 seconds, the test stimulus was presented for 5 seconds, and this was repeated twice. Figure 1B shows the presentation sequence. Perceived brightness of each test visual stimulus was quantified using the reference visual stimulus (visual stimulus(C) at highest luminance) set at 100. The participants’ responses were taken 3 seconds after stimulus presentation, considering the time constant of melanopsin. Each test session lasted approximately 20 minutes and was repeated three times. We set a 5-minute break in the dark between each trial. The mean value of repeated measurements of each experiment participant was used as experimental data. We applied a t-test (two-tailed) to elucidate statistical differences between the perceived brightness data.

### Pupil light reflex

Pupil diameter measurements were performed under each visual stimulus used in the brightness perception experiment. The measurements were obtained with the same participants, and the experiment was conducted in the same way as the brightness perception experiment. For pupil diameter measurement, a ViewPoint EyeTracker (Arrington Research, Inc., Arizona, USA) was used for both eyes. Measurements were obtained after resting in the dark for 5 minutes. The pupil diameter was measured in the dark for 10 seconds. Next, the pupil diameter under stimulation for 5 seconds was derived. We set 45-second intervals after each measurement. Figure 1C shows the presentation sequence.

## Results

### Brightness perception as a function of corneal luminance

We measured brightness perception under corneal luminance modulation for seven types of visual stimuli with different stimulus intensities on melanopsin at the same luminance. The metamerism method was used for separating the signals of melanopsin from those of cones and rods. A magnitude estimation method was used for quantifying perceived brightness for the presented visual stimuli.

First, the brightness perception measurement was conducted under the luminance modulation of visual stimuli (A) and (B) for verifying the involvement of melanopsin. Statistical tests were conducted to investigate the difference in brightness perception for the first (112 cd/m^2^) and second (102 cd/m^2^) highest luminance condition. Consequently, the P values of the perceived brightness between (A) and (B) stimulus were 0.002 for both luminance conditions, which indicates that the results can be explained by melanopsin contribution.

**Figure 3:**
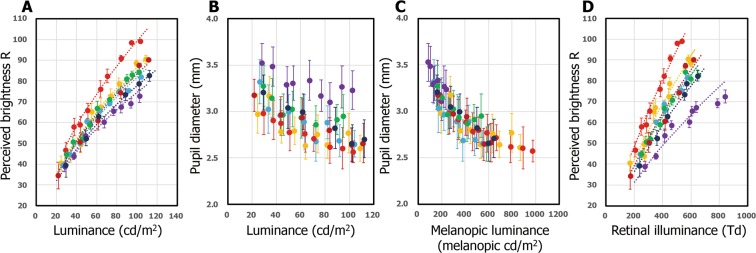
Brightness perception and pupil diameter under visual stimuli. (**A**) Perceived brightness as a function of corneal luminance. The participants’ responses were taken 3 seconds after stimulus presentation, considering the time constant of melanopsin. The colored circles represent the experimental data, broken lines represent best-fitted power functions. (**B**) Pupil diameter plotted as a function of corneal luminance. (**C**) Pupil diameter plotted as a function of melanopic luminance. (**D**) Perceived brightness as a function of retinal illuminance. The colored circles represent the experimental data, broken lines represent best-fitted power functions. Error bars represent standard errors of the mean. Each color corresponds to each visual stimulus in Fig. 2.

Second, brightness perception measurement was conducted under the luminance modulation of visual stimuli (C), (D), (E), (F), and (G) for formulating the perceived brightness for the visual stimulus on cones and melanopsin. Figure 3A shows the experimental results, including the data of the visual stimuli (A) and (B). Considering the image forming pathway involving the conventional cones, perceived brightness should be equal under the same luminance conditions, because the stimulus intensities on each L-, M-, and S-cone are constant. The results, however, showed that perceived brightness depends on visual stimuli presented under the same luminance conditions. A difference of approximately 1.4 times perceived brightness was noted at maximum.

### Measurement of pupil diameter and brightness perception as a function of retinal illuminance

It has been established that melanopsin signals are involved in the pupillary light reflex^15^. Therefore, even if a visual stimulus with an equi-luminance is presented, the pupil diameter may change when the melanopsin stimulus intensities are different. For clarifying the relationship between brightness perception and stimulus intensity on the retina, the pupil diameter under each stimulus was measured and the magnitude of visual stimulus was converted from luminance (cd/m^2^) to retinal illuminance (Td). The pupil diameter was measured using the same visual stimuli that were used for the brightness perception experiment. The pupil diameter in the dark before the presentation of stimuli and the minimum pupil diameter during the 5-second presentation were quantified. The results are shown in Fig. 3B. In addition, Fig. 3C is a replotted graph with the horizontal axis of melanopsin stimulus intensity. Figure 3D shows the relationship between brightness perception and retinal illuminance of each visual stimulus. Even at the same retinal illuminance, magnitudes of perceived brightness were noted differ. Remarkably, we found that the maximum difference in brightness magnitude was 1.8 times at the same retinal illuminance of 500 Td. The difference of perceived brightness in retinal illuminance is larger than that at the same luminance value, which suggests that our experimental results are not artifactual involving the pupil diameter, and that melanopsin significantly contributes to brightness perception.

### Formulation of brightness perception as a function of melanopsin

We formulated our experimental results for clarifying quantitatively the contribution to brightness perception of the stimulus intensity on melanopsin. The variables relating to the stimuli on the cornea and on cones of the retina were taken as the corneal luminance value (*L* cd/m^2^) and the retinal illuminance (*E* Td), respectively, represented by formulae (1) and (2). The variables relating to the stimuli on melanopsin (*M*: on the cornea, *G*: on melanopsin of the retina) were defined as the product of the melanopsin spectral power sensitivity *i*(*λ*) and spectral power distribution of visual stimulus *P*(*λ*) as the integral value over the entire wavelength range multiplied by the pupil area (*S*), represented by formulae (3) and (4).

1 $$L=683\int V(\lambda )P(\lambda )d\lambda $$

2 $$E=L\cdot S$$

3 $$M=4557\int i(\lambda )P(\lambda )d\lambda $$

4 $$G=M\cdot S$$

The values of *M* and *G* were calculated using the melanopic intensity proposed by Enezi *et al*.^6^ and Brown *et al*.^23^, where the *M* (melanopic luminance) ranged from 87 to 975 melanopic cd/m^2^ and *G* (melanopic retinal illuminance) ranged from 854 to 5054 melanopic cd/m^2^·mm^2^. We can derive the relationship between brightness perception and the stimulus intensity on melanopsin at the retina using Fig. 3D. By sorting all the data obtained by retinal illumination and fitting as a function of melanopic retinal illuminances, the following formula of the perceived brightness *R* can be obtained as a function of *G*.

5 $$R=4.84\cdot {10}^{-3}\cdot {G}^{1.1}+C$$

Here, *C* is a term associated with brightness perception by cones. Interestingly, the power number of the formula is nearly 1.0, which suggests that melanopsin directly contribute to brightness perception for obtaining the absolute brightness information. Concerning the integration of signals from cones, rods, and melanopsin, several studies exist on pupillary light reflex. Lucas *et al*. revealed a complementary role for the relation among cones, rods, and melanopsin in sum over a wide luminance range, which disappeared in the high luminance region in melanopsin knockout mice^34^. In addition, Tsujimura *et al*. indicated that it was a non-linear sum^28^ or a negative interaction^35^ in human. Therefore, we adopted a model where the relationship between melanopsin and cones in brightness perception is a sum. By fitting all experimental data with formula (5), we formulated brightness perception as a function of the stimulus intensity on melanopsin and that on cones.

6 $$R=4.84\cdot {10}^{-3}\cdot {G}^{1.1}+2.31\cdot {E}^{0.48}$$

The coefficient of determination, *r*^2^, showing the correlation between the calculated values and the experimental values was 0.94. The perceived brightness can be expressed by the summation of the term of melanopsin and the term of cones using power functions, which indicates that the signals from the two photoreceptor cells are complementary. We thus set the perceived brightness of the reference stimulus as 100 in this study.

Current photometry necessitates the derivation of a formulation for the stimulus at the cornea. We obtained the following formulae among the pupil diameter, the stimulus intensity on melanopsin and cones at the cornea from Fig. 3B, C.

7 $$d=1.74/(1+{e}^{(0.0040\cdot M+0.0012\cdot L)})+2.58$$

The coefficient of determination, *r*^2^, showing the correlation between the calculated values and the experimental values was 0.85. Recent studies have shown that roles of melanopsin, L-cone and M-, S-cone in pupillary reflex differ^36–38^. Our study showed that stimulus on melanopsin and luminance were excitatory to pupil light reflex.

The brightness perception with the stimulus intensity on cones and melanopsin at the cornea can be formulated as follows.

8 $$R=4.84\cdot {10}^{-3}\cdot {(M\cdot {\rm{\pi }}\cdot {(d/2)}^{2})}^{1.1}+2.31\cdot {(L\cdot {\rm{\pi }}\cdot {(d/2)}^{2})}^{0.48}$$

**Figure 4:**
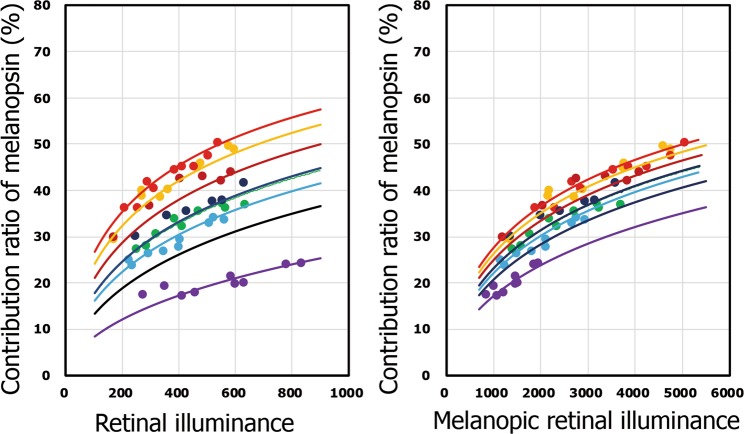
Contribution ratio of melanopsin in brightness perception. Contribution ratio of melanopsin is plotted as a function of retinal illuminance (left) or melanopic retinal illuminance (right). Solid lines indicate the extrapolation of the calculated results. Each color corresponds to each visual stimulus in Fig. 2. Black solid line indicates the calculated result in the case of commercially available white LED whose M/P ratio is 4.2 (see discussion).

The coefficient of determination, *r*^2^, showing the correlation between the calculated values and the experimental values was 0.95. Figure 4 shows the relationship between the ratio of melanopsin contribution to brightness perception and the stimulus intensity. The melanopsin contribution to brightness perception was found to increase to about 50% with increasing visual stimulus.

## Discussion

We used visual stimuli that modulated the extensive melanopic luminance at the same luminance by designing the spectral power distributions with white light sources and evaluated the perceived brightness directly using visual stimuli with modulated luminance. We obtained three findings in the present experiment. First, perceived brightness differs, even with the same luminance or the same retinal illuminance, if the stimulus intensity on melanopsin is different over a luminance range. Statistical tests were conducted to investigate the difference in brightness perception between stimuli C (M/P ratio 9.3) and E (M/P ratio 5.6), between stimuli C and G (M/P ratio 2.6), and between stimuli E and G for 102 cd/m^2^. In any case, the P values were less than 0.01 (Bonferroni-corrected). In addition, the determination coefficient between the experimental value and the calculated value was 0.57 when formulated only with the cone term. This is greatly inferior to the determination coefficient (0.94) when formulated with the sum of the melanopsin term and the cone term. This clearly demonstrated that melanopsin are involved in brightness perception. Brown *et al*. revealed the relationship between the test radiance at equal brightness and the melanopic excitation of the reference stimulus^23^. Estimated from the results of the present study, the test radiance at the same brightness with +11% melanopic excitation is +13%, which indicates that our results are also consistent with their data. Second, the formulated brightness perception can be expressed by the summation of linear terms associated with melanopsin and non-linear terms associated with cones, and the power number for stimulus on melanopsin was found to be nearly 1.0. In the visual information processing mechanism involving cones, the visual stimulus information encodes spatiotemporal contrast information via receptive fields. In addition, cones are easily adapted by light. Therefore, absolute brightness information relating to the light environment is not acquired by cones. The relation between the visual stimulus intensity for melanopsin and the perceived brightness is linear, which suggests that the absolute brightness information may be coded under relatively limited light adaptation of melanopsin. Thus, melanopsin appear to determine the perceived brightness level, and cone responses are added to it. In the non-image forming pathway, circadian rhythm photo-entrainment is performed by projecting from ipRGCs to the SCN. In this projection pathway, absolute intensity information regarding the light environment may be also necessary for controlling biological functions. Third, the contribution ratio of melanopsin in brightness perception was quantified. To our knowledge, this the first study that demonstrates the contribution ratio of melanopsin in brightness perception. As shown in Fig. 4, the contribution ratio of melanopsin increases with increasing visual stimulus intensity. Since the response to stimulus on melanopsin is irradiance-dependent (luxotonic)^25^, it is reasonable that the melanopsin’s ratio of brightness perception increases with increasing stimulus.

Zele *et al*. proposed analytical parameters for a brightness model from experiments using monochromatic LEDs^39^. They showed the following two components: additive and subtractive, and wavelength dependent coefficients regarding the cone contribution. In this study, however, we precisely maintained each stimulus intensity on L-, M-, and S-cones intact at the same luminance or the same retinal illuminance conditions and clearly showed that brightness perception of complex lights can be explained by a simple summation of a linear term of melanopsin and a non-linear term of cones with only positive coefficients. We derived the formulation using a step-by-step analysis, but not by an ad hoc procedure. In addition, our model can explain the fact that there is a brightness perception even in blind people lacking the function of cones and rods, because *R* has a positive value when *G* (melanopsin) is a positive value even if *E* (cones) is zero. Therefore, our model is quite reasonable.

In the present experiment, the duration time was 5 seconds, and the perceived brightness was responded at 3 seconds after stimulus onset was measured. The contribution ratios of melanopsin to the brightness perception (Fig. 4) were values at this moment. However, studies on pupillary light reflexes showed that the relative contributions of cone, rod and melanopsin responses vary with visual stimulation time^40^. Therefore, such contribution ratios may be subject to change of presentation time.

Brightness perception may be modulated by rod responses. For efficiently acquiring the signal from melanopsin, visual stimuli were presented to the peripheral visual field which is also an area where rods are distributed at high density. In this study, we eliminated the influence of rods as follows: 1) The luminance range in the experiment was over the region where the saturation of the rods is advanced. 2) A statistically significant difference (P = 0.002) was derived in brightness perception between visual stimuli having the same stimulus intensity on cones and rods but different stimulus intensity on melanopsin. 3) We formulated as function of stimulus intensity on cones and that of melanopsin using visual stimuli (C), (D), (E), (F), and (G). The visual stimuli (C), (D), (E), (F), and (G) correlate with the stimulus intensity on melanopsin and that on rods. However, the visual stimulus (A) has no correlation with them. If we can explain the data of visual stimulus (A) and (B) using the above formula, it can be said that this formula shows the involvement of melanopsin. We applied visual stimuli (A) and (B) individually to the results of formulation. Since both of the determination coefficients for the correlation between the calculated value and the experimental value were 0.95, it can be regarded as involvement of melanopsin, suggesting that rods are not involved in brightness perception under these conditions. Therefore, we concluded that this result was because of the involvement of melanopsin.

Because of the absorption of light at the crystalline lens and the vitreous body, the spectral power distributions of the light reaching the retina are different from those of the light sources. Therefore, the actual spectral power distribution reached at the retina may be different, depending on individuals. In this experiment, however, participants were young people in their twenties. Compared with elderly people, young people have less influence of age-related changes of crystalline lens and vitreous body dysfunction. If brightness perception targeting a wide range of age groups is formulated, correction of spectral power distributions would be required. We confirmed that there was no change in the relative relation in L-, M-, S-cone and melanopsin stimulus intensity among each visual stimulus between the ages of 32 and 25 under the change of transmittance of the crystalline lens with age using the model proposed by Pokorny *et al*.^41^. In addition, we used visual stimuli with a finite size for peripheral vision, a specific luminance range and only one chromaticity condition. Therefore, future research should be conducted on brightness perception using a wider field of vision, a wider range of brightness, and various hues other than achromatic color.

It may be wondered why such large contributions of melanopsin to brightness perception have not been previously identified. In the present study, we created artificial visual stimuli with a wide range of intensities on melanopsin using six-primary-color projectors and quantitatively compared the effect of melanopsin as a function of stimulus intensity in brightness perception. The M/P ratio ranged from 9.3 to 2.6 (Fig. 2). Conversely, the M/P ratio of commercially available light sources such as fluorescent lamps and white LEDs (5000 K) are 3.8 and 4.2, respectively. Contrast of M/P ratio was maximum 3.6. In the similar previous study^23,29^, the contrast of melanopsin at the cone silent was 1.22 or 1.148 respectively. We believe that we could separate and quantify the contribution of melanopsin in brightness perception with the large contrast. Therefore, we were able to clarify the actual contribution ratios of melanopsin in brightness perception.

The spectral sensitivity curve of melanopsin is influenced by macular pigment density. The position of the visual stimulus in this experiment may be affected by the macular pigment. We also examined the case using sensitivity curves of melanopsin, as proposed by Tsujimura *et al*.^42^ considering these factors. The result of formulation was almost the same as formula (6). The coefficient of determination, r^2^, showing the correlation between the calculated values and the experimental values was 0.95.

In conclusion, we formulated brightness perception involving melanopsin and cones. Furthermore, the contribution ratios of melanopsin and cones for brightness perception were clarified. Perceived brightness in humans can be determined by a summation of a linear term of melanopsin and a non-linear term of cones with positive coefficients not depending on the wavelength of light stimulus. Consequently, the conventional photometry system must be upgraded as brightness perception should be expressed by three dimensions (brightness vs. luminance and stimulus intensity on melanopsin) not two dimensions (brightness vs. luminance).

## Supplementary information

Supplementary information

## Data Availability

The dataset generated and analyzed during the current study are available from the corresponding author on reasonable request.
