# Supplementary material for: A quantitative analysis of the contribution of melanopsin to brightness perception
Source: Sci Rep. 2019 May 20;9:7568. doi: 10.1038/s41598-019-44035-3 (PMC6527610; doi:10.1038/s41598-019-44035-3)
Supplement: Supplementary file 1 — Supplementary information [file 41598_2019_44035_MOESM1_ESM.docx]

A quantitative analysis of the contribution of melanopsin to brightness perception

Masahiko Yamakawa^1*^, Sei-ichi Tsujimura^2^ and Katsunori Okajima^3^

^1^Graduate School of Environment and Information Sciences, Yokohama National University, Japan

^2^Department of Information Science and Biomedical Engineering, Kagoshima University, Japan

^3^Faculty of Environment and Information Sciences, Yokohama National University, Japan

Corresponding author: Masahiko Yamakawa

Correspondence to yamakawa-masahiko-wc@ynu.jp


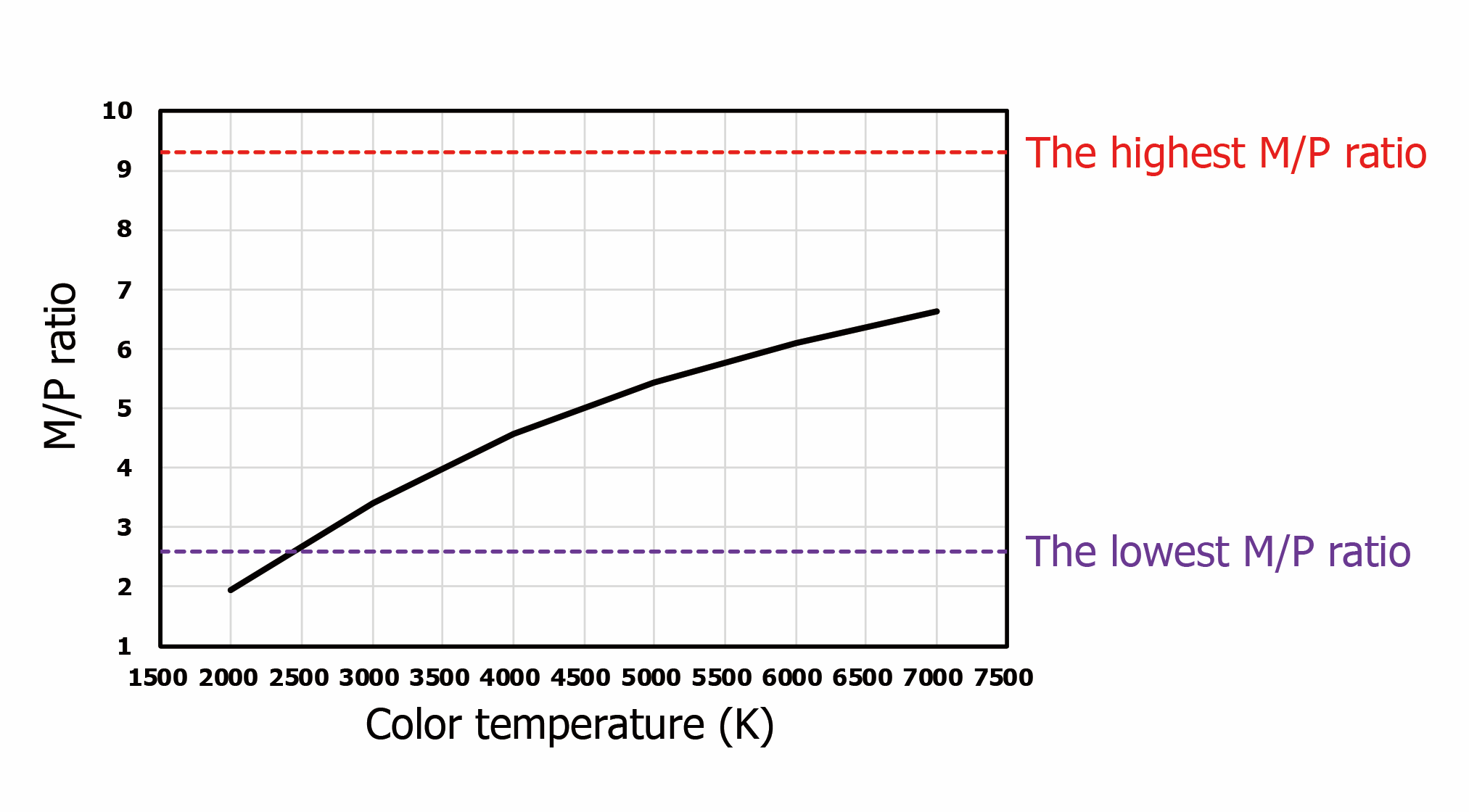


Figure S1. The M/P ratio of “Daylight” from low color temperature (2000K) to high color temperature (7000K) in the real world.

The M/P ratios of daylight cannot exceed the highest M/P ratio (9.3) used in this experiment. Therefore, such high M/P contrasts we set in the present experiment would be substantially less in the real world.
